# Comparison of bison and elk susceptibility to experimental challenge with *Brucella abortus* strain 2308

**DOI:** 10.3389/fvets.2024.1519453

**Published:** 2025-01-15

**Authors:** S. C. Olsen, P. M. Boggiatto, E. J. Putz

**Affiliations:** Infectious Bacterial Diseases of Livestock Research Unit, National Animal Disease Center, Agricultural Research Service, United States Department of Agriculture, Ames, IA, United States

**Keywords:** bison (*Bison bison*), elk (*Cervus canadensis*), *Brucella abortus* 2308, *Brucella*, pathogenesis

## Abstract

**Introduction:**

Brucellosis is endemic in bison and elk in Yellowstone National Park and surrounding areas.

**Methods:**

A comparative study was conducted using data from naive (*n* = 82 and 67, respectively) and *Brucella abortus* strain RB51 (RB51) vaccinated (n-99 and 29, respectively) bison and elk experimentally challenged with virulent *B. abortus* strain during pregnancy.

**Results:**

The incidence of abortion, fetal infection, uterine or mammary infection, or infection in maternal tissues after experimental challenge was greater (*p* < 0.05) in naïve and vaccinated bison when compared to similar groups in elk. Vaccinated bison had lower (*p* < 0.002) abortion rates and recovery of *Brucella* from fetal or uterine/mammary tissues when compared to naïve bison. Vaccinated elk had reduced (*p* < 0.01) rates of maternal infection, but rates of abortion and fetal or uterine/mammary infection did not differ (*p* > 0.05) from naïve elk. Naïve and vaccinated bison had greater (*p* < 0.05) *Brucella* colonization in placentomes, and parotid and supramammary lymphatic tissues when compared to elk. In elk or bison that aborted, mean colonization in placentome tissues were typically more than 5 logs higher than in animals that did not abort.

**Discussion:**

The results of our study suggest differences in disease pathogenesis between these two wildlife reservoirs of *B. abortus*.

## Introduction

Although elimination of *Brucella abortus* from cattle was essentially achieved in most of the United States by the early 2000s, the maintenance of the disease in wildlife reservoirs in the states of Idaho, Montana, and Wyoming has impaired the total eradication of brucellosis from cattle. In those states, free-ranging bison (*Bison bison*) and elk (*Cervus elaphus nelsoni*) have been known for many years to be infected with brucellosis (1917 and 1930, respectively) (1, 2). Management of the two species for brucellosis control differs as free-ranging bison predominantly reside within Yellowstone National Park and elk are more widely distributed. It should also be noted that elk populations in the area are greater and more widespread than bison, with disease control measures impacted by the congregation of elk on 22 states and 1 federal winter feed grounds (3). Both wildlife reservoirs in this area maintain high seroprevalence for brucellosis (historically approximately 35% for female elk on feed grounds and 50–60% for bison in Yellowstone National Park) (3–5), and disease prevalence in elk appears to be expanding outward at a rate of 5–8 km/year (6–8). Historical and epidemiologic data suggest that initial infection in these wildlife hosts resulted from lateral transmission of brucellosis from domestic livestock.

During winter months, the migration of bison out of Yellowstone National Park and the movement of elk to lower elevations can lead to interactions with domestic livestock, resulting in the transmission of brucellosis. In the areas surrounding Yellowstone National Park, state and federal agencies maintain vaccination policies and serologic testing for all animals grazing in areas with high brucellosis seroprevalence in wildlife. Despite expensive control measures, disease transmission from wildlife reservoirs has resulted in the identification of approximately 2.5 infected cattle herds each year in the region for the last decade. At the present time, all new infected cattle herds have been epidemiologically linked to the transmission of disease from free-ranging elk (9, 10). Despite efforts to prevent wildlife-cattle interactions during feeding during winter months, epidemiologic implications that elk are the source of infection may suggest that current management practices to maintain temporal and spatial segregation between domestic livestock and bison are effective.

Billions of dollars have been spent on the brucellosis eradication program in the United States since its inception in the 1930s as a drought recovery program (11, 12). Current disease control efforts in the Greater Yellowstone Area are expensive and require long-term commitments as they primarily target domestic livestock and do not reduce disease prevalence in wildlife reservoirs. Improving control of brucellosis in the area will require initiating efforts to reduce disease prevalence in free-ranging wildlife reservoirs. We have previously demonstrated phenotypic differences in susceptibility to brucellosis between bison and cattle (13), and also reported that elk fail to develop cellular immune responses after brucellosis vaccination (14). To provide basic data needed for the development of successful intervention strategies, we conducted a meta-analysis of data from multiple challenge studies to document phenotypic differences between naïve and *B. abortus* strain RB51-vaccinated bison and elk after experimental infection with *B. abortus* strain 2308 during gestation. These data demonstrate immunologic and pathophysiologic differences between the two species, emphasizing the need for infectious disease research to be conducted in the species of interest. Some of these data have been previously presented in other manuscripts (15–19).

## Materials and methods

### Vaccination

For vaccination, elk and bison were vaccinated intramuscularly with 1–6 × 10^10^ colony-forming units (CFU) of *B. abortus* strain RB51 (RB51) between 4 and 12 months of age. For this study, data from naïve or single-vaccinated bison and elk from independent efficacy studies (17 and 8, respectively) were compared [(15–18, 20–22) and unpublished data].

### Breeding and experimental challenge

Bison and elk heifers were obtained from brucellosis-free herds and housed at the National Animal Disease Center in Ames, IA, during the study. All animals were pasture-bred at approximately 2 1/2 years of age. Pregnancy status and stage of gestation in bison were determined by rectal palpation. In elk, pregnancy was confirmed by serologic measurement of pregnancy-specific protein B. Pregnant bison and elk were transferred to a biolevel 3 containment facility 2 to 3 weeks prior to experimental challenge where they were housed during the study. Bison and elk were experimentally infected with 10^7^ CFUs of *B. abortus* strain 2308 administered bilaterally to conjunctival surfaces (50 μL per eye) at an estimated 12 weeks prior to parturition. Data were also included from two studies in which additional pregnant elk were experimentally challenged with 10^8^ CFUs of *B. abortus* strain 2308 at a similar time in gestation. Concentrations of viable bacteria within each challenge were determined by serial dilution in saline and standard plate counts.

Bison and elk were euthanized within 1 week of the anticipated parturition date or within 72 h of abortion or parturition by intravenous injection of pentobarbital sodium. Fetal viability was assessed in both elk and bison by calf mobility and demonstration of milk in the stomach at necropsy. Maternal samples obtained at necropsy for bacteriologic evaluation using sterile forceps and a scalpel included lymphatic tissues (bronchial, hepatic, internal iliac, mandibular, mesenteric, parotid, prescapular, retropharyngeal, and supramammary lymph nodes), samples of milk and mammary tissue from all four quarters, maternal placentome, spleen, liver, lung, and vaginal and conjunctival swabs. Fetal samples included: rectal swab, lung, liver, spleen, bronchial lymph node, and abomasal contents. Fetal and maternal blood was also obtained at necropsy for bacteriologic evaluation.

Abortion was defined as the premature birth of a *Brucella*-infected, non-viable fetus at any time after experimental challenge with *B. abortus* strain 2308. Fetal infection was defined as bacteriologic recovery of the challenge strain from any fetal sample. Mammary/uterine infection was defined as the bacteriologic recovery of the 2308 challenge strain from milk, mammary gland, supramammary lymph node, placentome, vaginal swab, or any fetal sample. Evaluation of uterine/mammary infection allows assessment of potential transmission as transfer of infection occurs laterally through fluids or tissues associated with the abortion/parturition process (most common) or vertically through milk to the offspring (less common). Maternal infection was defined as the recovery of the 2308 challenge strain from any maternal sample. The time between experimental challenge and parturition was calculated for both vaccinated and naïve bison and elk.

### Bacterial culture

For bacterial culture, the tissues were macerated in 0.85% NaCl (saline) using a tissue grinder and placed on tryptose agar containing 5% bovine serum or Kuzdas and Morse agar as previously described (17, 23). The swabs were directly plated on the media. Following incubation at 37^o^ C and 5% CO_2_, *B. abortus* was identified by colony morphology and growth characteristics (24), and isolates were confirmed by a polymerase chain reaction procedure using primers specific for identification of *B. abortus* omp2a (25).

In 6 of the elk and 12 of the bison studies, colonization (CFU/gm) of *B. abortus* in four target tissues (supramammary, parotid, and prescapular lymph nodes and placentomes) was determined by obtaining a cross-section of the tissue, weighing the sample (approximately 1 gm), macerating the sample in a tissue grinder, preparing serial dilutions in saline, and placing aliquots in duplicate on media. Dilutions of the tissue homogenate extended to 10^−9^. Estimated concentrations of bacteria within tissues were determined by performing standard plate counts.

### Statistical analysis

Bacteriologic data are presented as mean ± SEM. Colonization (CFU/gm) data were converted to a logarithmic scale for analysis. Any values of zero were converted to 1 prior to logarithmic conversion. Colonization data and time between challenge and parturition were compared using the generalized linear model (SAS Institute Inc., Cary, NC, United States) and presented as mean ± SEM. Fisher’s exact test was used to compare the incidence of abortion and infection between naïve or vaccinated bison and elk following experimental challenge.

## Results

For the study, data were analyzed from 82 naïve and 99 RB51-vaccianted bison and 67 naïve and 29 vaccinated elk. Of the 67 naïve elk, 52 were experimentally challenged with 10*^7^* CFU and 15 were infected with 10^8^ CFU of *B. abortus* strain 2308. Statistical analysis demonstrated that pregnant elk challenged at approximately 12 weeks prior to parturition with either 10^7^ or 10^8^ CFU of strain 2308 did not differ in rates of abortion (*p* = 0.09), fetal infection (*p* = 0.95), uterine/mammary infection (*p* = 0.78), or maternal infection (*p* = 0.19) (Table 1). In addition, the time between experimental challenge and parturition did not differ (*p* > 0.05) between elk infected with high or low challenge dosages. As there were no statistical differences between these two groups, both groups were combined for subsequent statistical analyses.

**Table 1:** Abortion and infection rates after conjunctival infection of naïve elk with two dosages of *B. abortus* strain 2308 during gestation.

	N	Abortions	Fetal infection	Uterine/mammary infection	Maternal infection
10^7^ CFU	52	3 (6%)	10 (19%)	29 (56%)	20 (39%)
10^8^ CFU	15	3 (20%)	3 (20%)	6 (40%)	12 (80%)

### Species differences in susceptibility to abortion and infection and differences in vaccine efficacy

When abortions and infections were compared between naïve bison and elk, naïve bison had greater (*p* < −0.001) rates of abortion, fetal infection, uterine/mammary infection, and material infection after experimental challenge with *B. abortus* strain 2308 during gestation (Table 2). Similarly, RB51-vaccinated bison had higher abortion rates (*p* < 0.01) and greater rates of infection (*p* < 0.025) in fetal, uterine/mammary, or maternal tissues than vaccinated elk. In vaccinated bison, there was a reduction in abortion rates (*p* < 0.002) and recovery of *Brucella* from fetal or uterine/mammary tissues (*p* < 0.001) as compared to experimentally infected naïve bison. Vaccinated elk had reduced (*p* < 0.01) rates of maternal infection, but rates of abortion and fetal or uterine/mammary infection did not differ (*p* > 0.05) from naïve elk after experimental challenge.

**Table 2:** Abortion and infection rates after mid-gestational challenge of naïve or RB51 vaccinated bison and elk with *Brucella abortus* strain 2308 during gestation.

	N	Abortions	Fetal infection	Uterine/mammary infection	Maternal infection
Naive bison	82	69 (84%)*^†^	69 (84%)*^†^	72 (88%)*^†^	81 (99%)*^†^
Elk	67	6 (9%)	13 (19%)	29 (43%)	44 (66%)^‡^
RB51 vaccinates bison	99	36 (36%)*	52 (53%)*	54 (55%)*	84 (85%)*
Elk	29	3 (10%)	4 (14%)	7 (24%)	11 (38%)

Abortion or infection rates of bison denoted with “*” were greater (*p* < 0.01) than abortion or infection rates of elk. Abortion or infection rates of naïve bison denoted with “†” were greater (*p* < 0.001) than abortion or infection rates of vaccinated bison. Maternal infection rates of naïve elk denoted with “‡” were greater (*p* < 0.001) than maternal infection rates of vaccinated elk.

### Lack of species differences in gestation length after experimental infection

The time between challenge and parturition in naïve and vaccinated elk did not differ in animals that delivered full-term calves (111.6 ± 3.7 and 123.3 ± 3.5 days, respectively). Similarly, no differences (*p* > 0.05) between challenge and parturition were detected for full-term calves from naïve and vaccinated bison (75.3 ± 7.2 and 85.6 ± 2.7 days). Recognizing the low percentage of elk that aborted, there was a shorter time (*p* < 0.05) between experimental challenge and abortion as compared to full-term calves in naïve (81.0 ± 20 and 112.3 ± 3.5 days, respectively) and vaccinated elk (77.7 ± 1.4 and 92.8 ± 14.8 days, respectively). The time between experimental challenge and parturition did not differ (*p* > 0.05) between naïve and vaccinated bison that aborted (46.4 ± 1.4 and 51.4 ± 2.1 days, respectively), but the length of time in both groups was shorter (*p* < 0.05) when compared to animals delivering full-term calves.

### Species differences and vaccine effects on *Brucella* colonization

In naïve bison and elk, colonization in placentome and parotid, prescapular, and supramammary lymph nodes was greater (*p* < 0.05) in animals that aborted as compared to animals that delivered full-term calves (Table 3). In naïve bison and elk that delivered full-term calves, colonization in parotid, prescapular lymph node and placentome was greater (*p* < 0.05) in bison. Recognizing the low number of elk that aborted, only colonization in the placentome differed (*p* < 0.05) between naïve bison and elk after abortion. In vaccinated animals, bison had higher colonization (*p* < 0.05) in placentome, parotid, and supramammary tissues than elk after delivery of a full-term calf. In vaccinated animals that aborted, colonization in placentomes of bison was greater (*p* < 0.05) than mean bacterial numbers recovered from elk samples. This observation may have been influenced by the low number of vaccinated elk that aborted. Of epidemiologic importance is the approximately 5-log reduction in both naive and vaccinated bison between animals that delivered full-term calves as compared to those that aborted. A similar difference in placentome colonization was noted between full-term and aborting naïve elk. In RB51-vaccinated elk, differences in placental colonization between full-term and aborting elk were only 2 logs but, as discussed previously, may have been influenced by low numbers of vaccinated elk that aborted in our studies.

**Table 3:** Colonization (CFU/gm) of target tissues (lymph nodes and placentomes) after mid-gestational challenge of naïve or RB51-vaccinated bison and elk with *Brucella abortus* strain 2308 during gestation.

		Parotid	Prescapular	Supramammary	Placentome
Naive					
Elk	Full term	0.77 ± 0.19^a^	0.12 ± 0.09^a^	0.37 ± 0.09^a^	1.05 ± 0.40^a^
	Abort	1.94 ± 0.19^bc^	1.66 ± 0.43^b^	2.14 ± 0.55^b^	5.95 ± 1.57^b^
Bison	Full term	1.56 ± 0.34^c^	0.73 ± 0.33^c^	0.77 ± 0.35^a^	2.92 ± 1.01^c^
	Abort	2.70 ± 0.12^b^	1.83 ± 0.13^b^	2.37 ± 0.17^b^	7.92 ± 0.23^c^
RB51 vaccinates					
Elk	Full term	0.12 ± 0.08^a^	0 ± 0^a^	0 ± 0^a^	0.22 ± 0.16^a^
	Abort	1.43 ± 0.65^abc^	1.30 ± 0.48^b^	1.89 ± 0.84^b^	2.14 ± 1.94^ab^
Bison	Full term	0.99 ± 0.19^b^	0.35 ± 0.11^ab^	0.77 ± 0.25^b^	1.98 ± 0.55^b^
	Abort	1.61 ± 0.17^c^	0.75 ± 0.16^b^	1.64 ± 0.22^b^	6.91 ± 0.60^c^

Means within a tissue within naïve or vaccinated treatments with different superscripts are statistically different (*p* < 0.05).

## Discussion

In a similar manner as noted previously when comparing cattle and bison, our data suggest differences in the pathophysiology of brucellosis between bison and elk after experimental infection. In general, bison abort at a higher rate and have higher infection rates in fetal, uterine/mammary, and maternal tissues when compared to elk. Vaccination of bison with RB51 reduces abortion rates after experimental infection when compared to naïve animals, but similar efficacy by vaccination was not noted in elk. As naïve elk had a low abortion rate after experimental challenge, no difference was detected in fetal wastage between vaccinated and naïve elk after experimental challenge. However, it should be noted that almost 70% of naïve elk had *Brucella* recovered from tissues at parturition, suggesting a high infection rate after experimental challenge. The higher abortion and infection rates in bison indicate that the species is more susceptible to brucellosis and its adverse effects on reproduction when compared to elk.

Abortion rates in elk in the current study were comparable to estimates of 6% fetal losses (CI: 2–15%) in seropositive elk under field conditions (26). The same study estimated that 8% (CI: 4–19%) of seropositive elk shed *B. abortus* after abortion or live birth under field conditions, which is lower than data from the current study. As the field study was conducted in seropositive elk greater than 2 years of age and primarily relied on culture data from an expelled vaginal implant transmitter, differences between the two studies could have been influenced by the acute nature and more extensive sampling in the experimental challenge studies, the length of time between initial infection and parturition, and other factors. Others have estimated a 31% reduction in pregnancies in 2-year-old elk and a 7% reduction in pregnancies in elk 3 to 9 years of age in a longitudinal study in seropositive elk on Wyoming feed grounds (27). Overall, experimental and field data suggest that abortion rates from brucellosis in elk are considerably less than those observed in bison and cattle.

The epidemiologic characteristics of brucellosis in the chronically infected Yellowstone and Grand Teton National Park bison herds have been evaluated. Although reports documenting abortions have been limited in the Yellowstone herd (28, 29), the herd has high seroprevalence (50–60%) with approximately half (46%) of seropositive bison found to be culture positive for *B. abortus* (28, 30). Although the population is smaller, higher seroprevalence (77%) has been reported in the Grand Teton herd as compared to the Yellowstone herd (31), but published data on abortion rates are lacking. Although recoveries from maternal tissues of bison were higher in our experimental studies, the higher rate may reflect evaluation of infection during more acute stages of disease. As exposure to virulent *B. abortus* field strains would be anticipated to induce immunologic responses comparable to responses after vaccination, it would be of interest to compare abortion rates in these chronically infected herds to vaccine efficacy data obtained under experimental conditions.

We observed that colonization (CFU/gm) in target tissues is somewhat similar between the two species but frequently is higher in bison when compared to the paired treatment in elk. The four tissues chosen for evaluation were selected because they represent lymphatic tissues at the site of experimental challenge (parotid lymph node), localization in reproductive tissues (placentome), mammary gland infection (supramammary lymph node), or a lymph node not associated with a preferred localization site for *Brucella* (prescapular lymph node). The abortion event is the most significant route of transmission of brucellosis for ruminants, and higher numbers of bacteria in the uterine environment are associated with greater capability for lateral transmission of brucellosis from expelled fluids, placental tissues, or fetal tissues. As a minimum infectious dosage of *B. abortus* is estimated to be between 10^3^ and 10^4^ CFU (32), the high concentrations (sometimes up to 10 logs of *Brucella* per gm) detected in placentomes at abortion in bison and elk demonstrate significant capability for lateral transmission. Our data demonstrated that vaccination reduces abortions by approximately 42% and average bacterial colonization increases by 5 logs of bacteria within placentomes of bison that abort. The combination of reduced abortions and reproductive colonization would be anticipated to reduce disease transmission under field conditions.

Our data suggested that co-localization in both fetal and reproductive tissues is common, but it is extremely rare to find bison or elk with *B. abortus* colonization only within mammary tissues after experimental challenge during gestation. Although vertical transmission appears to be of lesser epidemiologic importance, brucellosis can be transmitted to calves through infected milk. It has been postulated that oral exposure of brucellosis to calves can lead to latent infections. In cattle, it was estimated that 2.5% of heifer calves from seropositive dams are at risk for developing and transmitting brucellosis (33, 34). Other studies in cattle have reported that 1.3–5.4% of calves nursing from infected cows develop persistent seropositive responses (34, 35). Although data are lacking in elk and greater characterization is needed in bison, seroconversion was observed in 12% of calves from a *Brucella*-infected bison herd that were seronegative when first quarantined (36). Currently, the epidemiologic importance of transmission of brucellosis through milk to elk or bison calves under field conditions requires further characterization.

We have previously reported that bison take up to 22 weeks to clear the attenuated *B. abortus* RB51 vaccine strain (20, 37). We have recovered the vaccine strain from elk as late as 44 weeks after inoculation (Olsen, unpublished data), and others have recovered RB51 from elk at 34 weeks (19). Unpublished data from murine models in our laboratory indicated that *in vivo* clearance of virulent strains of *B. abortus* is longer than the approximately 8 to 12 weeks required to clear attenuated vaccine strains (38). Therefore, the recovery of *B. abortus* from a high percentage of non-aborting bison and elk at parturition in the present study may reflect that temporal requirements for *in vivo* clearance exceed those allowed by the experimental challenge model (target of 12 to 14 weeks between experimental challenge and parturition). Therefore, data on infection rates at necropsy in the current study should be interpreted in the context of species-specific temporal limitations for clearance of *Brucella*.

Previous studies in elk have demonstrated that humoral responses predominate after brucellosis or tuberculosis vaccination and antigen-specific cellular immune responses are lacking (14, 19, 39). Other studies have also reported that abortion rates did not differ between RB51 vaccinates and naïve control elk after experimental challenge (40, 41). Others have reported differences in disease pathogenesis between bison and elk. In comparative studies on susceptibility to foot and mouth disease (FMDV), elk demonstrated milder clinical signs and did not transmit disease to contact elk in contrast to more severe clinical disease in bison (42). These data highlight the fact that differences in disease pathogenesis may require species-specific intervention strategies.

Overall, our data suggest significant differences between bison and elk in the pathogenesis of brucellosis and species differences in vaccine protection that may impact disease control. In concurrence with others, our data suggested that abortion events caused by brucellosis in elk are less common when compared to bison and cattle. The low rate of abortions in elk would initially appear to be contradictory to epidemiologic data that almost exclusively link them to the transmission of disease to cattle herds in the Greater Yellowstone Area. The presence of higher populations of elk with greater potential for interactions with cattle in the GYA area is more likely to be responsible for the correlation of elk with brucellosis transmission. However, both wildlife reservoirs can transmit disease (10, 43) and if given equal access to cattle herds, bison would appear to be a greater risk for brucellosis transmission due to their higher abortion rates.

## Data Availability

The original contributions presented in the study are included in the article/Supplementary material, further inquiries can be directed to the corresponding author.
